# The Effects of Within- and Between-Group Competition on Trust and Trustworthiness among Acquaintances

**DOI:** 10.1371/journal.pone.0103074

**Published:** 2014-07-18

**Authors:** Guofang Liu, Chongde Lin, Ziqiang Xin

**Affiliations:** 1 Institute of Developmental Psychology, Beijing Normal University, Beijing, P. R. China; 2 School of Psychology, Beijing Normal University, Beijing, P. R. China; 3 Department of Psychology at School of Social Development, Central University of Finance and Economics, Beijing, P. R. China; George Mason University / Krasnow Institute for Advanced Study, United States of America

## Abstract

Several studies have indicated that between-group competition is a key stimulator of trust and trustworthiness. Another important but neglected type of competition may also affect trust and trustworthiness: within-group competition, especially competition among acquaintances. The present study investigated the effects of both within- and between-group competition on trust and trustworthiness, which were measured using an investment game played by acquaintances. We found that, compared to the participants' performance in the non-competition condition, when individuals were motivated to compete with their in-group members or the other groups for financial rewards, they demonstrated more trust. When individuals were motivated to compete with their in-group members, they exhibited lower trustworthiness than in non-competition and between-group competition. In addition, within-group competition decreased the trustor's payoff while both within- and between- group competition increased the trustee's payoff. Finally, we found that males trusted their group members more than females.

## Introduction

Trust and cooperation play an important role in social and economic development, and explaining trust and cooperation is also one of the most interesting and greatest challenges for evolutionary psychology and social sciences [1]-[6]. Many researchers have proposed that trust is the key to cooperation [7]–[9]. Several possible mechanisms, such as punishment and reputation, are used to explain the occurrence of trust as well as cooperation [10]–[15]. Recently, competition has been found to be a major factor affecting trust and trustworthiness [16], [17]. However, the influence of competition on trust and trustworthiness is still unclear, especially the influences of within- and between-group competition on trust and trustworthiness among acquaintances.

There is evidence that competition is an important factor in trust and trustworthiness. Researchers have investigated the effect of competition among individuals on trust and trustworthiness [16], [17]. In their competition conditions, trustors selected their partners on the basis of trustees' reputation information. So trustees have to compete for the trust of trustors. In the non-competition conditions, trustors interacted with randomly assigned trustees. It is found that participants in competition conditions performed higher levels of trust and trustworthiness than in non-competition conditions. These studies demonstrated the strong link between trust and competition. However, these researchers did not consider the influence of group context on human behaviors. Indeed, we usually interact with each other in the context of groups [18], [19]. In group contexts, the effect of competition among individuals on trust would be moderated by participants' group identification. For example, when two individuals who are from different groups are competing with each other, they may make self-interested decisions because the possibilities of repeated interactions are low. However, when two individuals who are from the same group are competing with each other, they may consider their partners' interests because the possibilities of repeated interactions are high. That is, the effect of competition on trust may be influenced by group context. In a group, two basic competitions may affect trust and trustworthiness: within- and between-group competition.

Although few studies have investigated the effects of within- and between-group competition on trust and trustworthiness, a number of studies focusing on cooperation can shed light on the effects of competition, especially between-group competition. By allocating additional rewards to members of better-performing groups, researchers have found that between-group competition can improve cooperation among group members [20]–[26]. In these studies, members within one group share the same interests and will suffer the same consequences for their actions. To overcome the other groups, group members have to cooperate with each other, thus between-group competition improves cooperation among members within one group. According to group selection theory, groups with more in-group altruistic behavior have more opportunities to survive when competing with other groups [27], [28], thus the members in a group will show more in-group cooperative behaviors than they usually do when they facing between-group competitions. Because our cooperation may be exploited, the belief that the partners will not take advantage of our benevolence is important to cooperation. That is, trust is the precondition of cooperation [7]–[9]. We hypothesize that between-group competition can improve trust and trustworthiness behaviors among group members.

Meanwhile, another ubiquitous competition may also affect trust and trustworthiness: within-group competition. Within-group competition happens in diversified conditions among both animals and human beings. For example, cubs fight for breast nursing, male animals contend for sexual rights, and humans compete for leadership. All of these situations involve within-group competition, in which individuals are negatively interdependent, i.e., one's success is another's failure [9], [29]–[32]. In these situations, within-group competition (or conflict) reduces the cooperation among group members [33], [34].

However, it is noteworthy that, in previous studies, within-group competition usually took place among group members who did not know each other before, which means that such within-group competition actually is interpersonal competition [35]. In this situation, an opportunity to build a good reputation is necessary for the cooperation among individuals [36]–[39]. Charness, Du and Yang [40] have found that the participants who are trustworthy in previous interactions will get more trust than the participants who have no trustworthiness information (it is similar to a kind of reputation). However, in a group consisting of acquaintances rather than strangers, the motivation to build a good reputation is possible unnecessary for maintaining the cooperation among group members because individuals may have known the reputation of each other. Therefore, we infer that interaction experiences among acquaintances can moderate the influence of within-group competition on trust and trustworthiness. If individuals believe the benevolence of their group members, they may place the group's profit ahead of their own and thus the within-group competition will not undermine trust and trustworthiness among acquaintances. However, if individuals doubt the benevolence of their group members, within-group competition may reduce trust and trustworthiness among acquaintances. To check this proposition, we investigate the effect of within- and between-group competition on trust and trustworthiness among acquaintances.

We conducted a three-phase investment game to answer this issue. The first phase of the investment game is the non-competition (NC) game, which aims to obtain the baseline of trust and trustworthiness among acquaintances. The second phase is a within-group competition (WGC) investment game, which aims to explore the effect of within-group competition on trust and trustworthiness among acquaintances. The third phase is a between-group competition (BGC) investment game, which aims to investigate the effect of between-group competition on trust and trustworthiness among acquaintances. As mentioned above, within-group competition may undermine trust and trustworthiness, whereas between-group competition may foster trust and trustworthiness; thus, our third-phase experiment also aims to remove the possible negative effect of within-group competition on participants' trust and trustworthiness.

## Method

### Ethics Statement

This study was approved by the local ethical committee of Central University of Finance and Economics. All participants gave written informed consent.

### Participants

Twenty-five college students (13 females) in an introductory psychology class participated in this experiment. Their mean age was 19.40, *SD*  = 0.87. All of the participants had known each other for 6 months, and they had never participated in any other experiments about trust or competition.

### Procedures

An investment game was used to investigate the participants' trust and trustworthiness. The original game, which was developed by Berg, Dickhaut and McCabe [41], requires participants to play either the role of trustor or that of trustee. The modified investment game proposed by Burks, Carpenter and Verhoogen [42] allows participants to play both roles of trustor and trustee in turn to provide both measurements of trust and trustworthiness. In the present study, the participants must play both roles.

Participants completed the experiment in a classroom and were 2 m away from one another. Before the experiment, the participants were introduced to the investment game but were informed of neither the competition condition nor how many rounds the game would run. Participants were also informed that they would be paid based on their payoffs in a random round of the experiment. In each round of the investment game, each participant is awarded 10 yuan (RMB) for showing up. The trustor invests X (0≤X≤10) yuan in the trustee. The investment X is then tripled and sent to the trustee. After received the tripled investment, the trustee returns Y (0≤Y≤3X) yuan to the trustor. The trustor's payoff equals 10-X+Y, and the trustee's payoff equals 10+3X-Y. The trustor's trust level is represented by X, and the trustee's trustworthiness level is represented by their return ratio, Y/3X (if trustor invests 0 Yuan, trustee's return ratio is 0).

In the beginning of each round of the investment game, all participants are trustors. They select their investment X and write it on a slip of paper that records their investment and their partner's return Y. After the trustors place the recording paper in an envelope, the experimenter collects and delivers the envelopes to these participants randomly and anonymously. Now, all participants serve as trustees and they are asked to respond to the investment they received. These trustees select their return Y on the corresponding investment and write it on the recording paper. These slips are then returned to the envelopes. The experimenter then collects and returns the envelopes to their initial players. In each round of the investment game, the participants record their investment and their partner's return when they are trustors and their partner's investment and their own corresponding return when they are trustees.

To test the effects of within- and between-group competition on trust and trustworthiness, we divided the experiment into three phases. The first phase is non-competition, which consists of three rounds. Next, within-group competition was introduced by allocating a doubled-payoff reward to the three participants in the class who obtain the highest payoff in the following rounds of the investment game. After five rounds of within-group competition, the participants were informed that there was to be a comparison between the participants' mean payoff in the current class and that in another class in the following rounds' game. Between-group competition was introduced by allocating 5-yuan extra rewards to each participant in the higher mean payoff class. The between-group competition investment game was also comprised of 5 rounds. In all rounds of the investment game, the participants did not know whom they were interacting with. Lastly, the payoff in the third round of phase one was randomly selected as the payoff standard. The participants' mean payoff was 16.08 yuan, *SD*  = 5.27. Because participants were paid based on their payoffs in the third round of phase one, we did not run the same investment in the other class. That is, there was no real between-group competition.

## Results

There were no significant differences between the participants' performance (investment, return ratio, and payoff) among the three- or five-round games in each condition (or phase) (*ps* >.10), see Table 1 for a fully description. Thus, we averaged their performance in each condition as the dependent variables.

**Table 1:** Participant's performance in thirteen rounds (*M*± *SD*).

Conditions	Rounds	Investment (Yuan)	Return ratio (%)	Payoff-trustor (Yuan)	Payoff-trustee (Yuan)
NC	One	5.28±2.48	.37±.20	10.76±3.54	19.80±5.64
	Two	5.80±2.66	.40±.19	11.24±4.25	19.36±6.38
	Three	6.08±3.00	.42±.29	11.68±4.32	20.48±8.28
WGC	Four	7.28±2.73	.34±.20	10.16±4.07	24.40±7.23
	Five	6.32±2.75	.33±.18	10.28±3.74	22.36±6.16
	Six	6.92±2.81	.28±.19	9.16±4.12	24.68±7.39
	Seven	6.20±2.96	.25±.18	8.84±4.00	23.56±7.77
	Eight	6.36±2.72	.30±.20	9.72±4.41	23.00±6.60
BGC	Nine	7.16±2.49	.39±.22	11.48±5.64	22.84±6.97
	Ten	7.68±2.54	.36±.17	10.56±4.51	24.80±6.83
	Eleven	6.48±3.08	.35±.25	11.40±6.06	21.56±6.31
	Twelve	7.08±2.81	.40±.27	11.72±5.32	22.44±6.95
	Thirteen	6.80±3.15	.36±.20	11.28±3.58	22.32±6.89

Note: Payoff-trustor  =  Participant's payoff when acting as trustors; Payoff-trustee  =  Participant's payoff when acting as trustees.

To investigate the effect of competition on participants' trust, we ran a repeated measurement ANOVA on the participants' investment in the three conditions. Because gender is a potential factor of trust [43], [44], thus we set participants' gender as between-subjects factor in our analyses. The results showed a significant effect of condition, *F* (2, 22)  = 6.96, *p*<.01, *η^2^* = .39. Post hoc tests showed that the participants' investment in the WGC (*M* = 6.62, *SD*  = 2.19) and BGC (*M* = 7.04, *SD*  = 2.26) conditions were significantly higher than that in the NC condition (*M* = 5.72, *SD*  = 2.06) (*ps*<.01), and there was no significant difference between the participants' investment in the WGC and BGC conditions (*p*>.10). That is, individuals trust their partners more when they are motivated to compete with members of their own group or the other group than when they have no motivation to compete with others. We also found a significant effect of gender, *F* (1, 23)  = 4.49, *p*<.05, *η^2^* = .16, the investment of females (*M* = 5.74, *SD*  = 2.15) was significantly lower than that of males (*M* = 7.24, *SD*  = 1.20), *p*<.05, which indicates that males trust their classmates more than females. There was no significant interaction between experimental condition and gender, *p*>.10.

To investigate the effect of competition on participant's trustworthiness, we conducted a repeated measurement ANOVA on the participants' return ratio in the three conditions with participants' gender as between-subjects factor. Results showed a significant effect of condition, *F* (2, 22)  = 6.81, *p*<.01, *η^2^* = .38. Post hoc tests showed that the participants' return ratio in WGC condition (*M* = 0.30, *SD*  = 0.13) was significantly lower than that in NC condition (*M* = 0.39, *SD*  = 0.17) (*p*<.01) and in BGC condition (*M* = 0.37, *SD*  = 0.15) (*p*<.01). There was no significant difference in the participants' return ratio between the BGC and NC conditions (*p*>.10). These results indicate that WGC is harmful to individuals' trustworthiness, but BGC is not. There were no significant effects of gender and interaction between experimental condition and gender, *ps* >.10.

To check the effect of competition on participants' payoffs, we first ran a repeated measurement ANOVA on participants' payoff-trustor (participants' payoff when acting as trustors) with gender as between-subjects factor. Results showed that participants' payoff-trustor varied significantly by condition, *F* (2, 22)  = 6.83, *p*<.01, *η^2^* = .38. Post hoc tests showed that participants' payoff-trustor in WGC condition (*M* = 9.63, *SD*  = 1.82) was significantly lower than that in NC condition (*M* = 11.23, *SD*  = 2.24) (*p*<.01) and in BGC condition (*M* = 11.29, *SD*  = 2.63) (*p*<.05). That is, compared with NC and BGC, WGC is harmful to the trustor's payoff. Neither the effect of gender nor the effect of interaction between experimental condition and gender was significant, *ps* >.10.

We also ran a repeated measurement ANOVA to test the effect of competition on participants' payoff-trustee (participants' payoff when acting as trustees) with gender as between-subjects factor. The results showed that participants' payoff-trustee varied significantly by condition, *F* (2, 22)  = 9.17, *p*<.01, *η^2^* = .46. Post hoc tests showed that participants' payoff-trustee in the WGC condition (*M* = 23.60, *SD*  = 3.87) and BGC condition (*M* = 22.79, *SD*  = 3.69) were both significantly higher than that in the NC condition (*M* = 19.88, *SD*  = 3.75) (*ps* <.01), and there was no significant difference between participants' payoff-trustee in the WGC and BGC conditions (*p*>.10). The results indicate that WGC and BGC can both improve the payoffs of the trustees. Neither the effect of gender nor the effect of interaction between experimental condition and gender was significant, *ps* >.10.

## Discussion

Trust, cooperation and competition are key research topics in psychology. Recently, researchers have become increasingly interested in the effect of competition on trust and cooperation [16], [17], [20], [25], [45]. The present study investigated the influences of within- and between-group competition on trust and trustworthiness among acquaintances. We found that within- and between-group competition both improved trust and that within-group but not between-group competition reduced trustworthiness. And within-group competition undermined the trustor's payoff while the within- and between-group competition both increased the trustee's payoff. To sum up, within- and between-group competition affects the trustor and trustee's behavior differently.

First, within-group competition cannot achieve a win-win for the trustor and trustee in the same group. We found that trustors invested more money to their partners in WGC condition than in NC condition, but trustees returned less in WGC condition than in NC condition. We also revealed the asymmetric effect of within-group competition on trustor's payoff and trustee's payoff: within-group competition undermines trustor's payoff but improves trustee's payoff. Studies about group processes indicated that when individuals are encouraged to compete with their group members for their own success, they exhibit higher levels of aggressiveness to their group members and the group's productivity is lower [29], [32]. We suggested that within-group competition activates the individuals' belief that my teammates are self-interested, leading to the negative effect of within-group competition on trust. To maximize their payoff in WGC condition, trustors have to not only invest more money to trustees but also receive a high return from trustees, while trustees have to return less to trustors. This is the benefit conflict between trustors and trustees. If trustors recognize that trustees have the motivation to return none of the money, rational trustors should invest no money. Surprisingly, we found that trustors in the WGC condition invest more money than in the NC condition. In our experiment, participants have no opportunity to build a good reputation to get future trust, thus the positive effect of within-group competition on trust may be caused by the participants' positive expectations on the benevolence of their group members. In this study, participants had known each other for 6 months, and they may have built well trust relationships with each other. In the investment game, to obtain highest trustor-payoffs, it is necessary that trustors invest all money and trustees return all money. In the present WGC condition, trustors may believe their partners' trustworthiness, thus they dare to invest, which caused the seemingly positive effect of WGC on trust. If participants are strangers, within-group competition may undermine trust.

Second, between-group competition may achieve a win-win between trustors and trustees in the same group. We found that trustees invested more money to their partners in BGC condition than in NC condition, and there was no significant difference between the return ratio of trustees in NC and BGC condition. And trustee's payoff in BGC condition was significantly higher than that in NC condition, and there was no significant difference between the payoff of trustees in BGC and NC condition. In this situation, neither the benefit of trustors nor that of trustees were impaired, thus between-group competition brings the opportunity for the trustor and trustee to achieve a win-win by practicing a high level of trust and trustworthiness. This point is consistent with the prediction of the group selection theory [27], [28]. However, there are other possible interpretations. First, according to social identification theory [18], [46]–[48], between-group competition can strengthen individuals' group identification; thus, individuals will have a stronger in-group preference. Therefore, individuals in the BGC condition will exhibit a higher level of trust and trustworthiness. Second, if group members have to cooperate to complete a task or when they suffer from similar consequences from their actions, groups can achieve more productive consequences [29], [32], [49]–[51]. In the BGC condition, the participants have to cooperate to achieve a high group payoff which may lead to a win-win between trustors and trustees in the same group.

Researchers have demonstrated that trust and cooperation is context-dependent. For example, trust level is affected by the events that individuals involved in [52] and the punishment opportunity to untrustworthy partners [53]. In daily life, individuals usually interacted (especially competed) with each other under group contexts. In these situations, trust and cooperation may be affected by between- and within- group competitions. It is revealed that between- and within-group competitions both improved participants' trust level, and within-group competition inhibits participants' trustworthiness.

Although the present study yielded valuable results, it is necessary to note that all assessments of the effects of between-group competition on trust, trustworthiness and payoffs are based on the fact that the participants' behavior has been affected by within-group competition in phase 2. This introduces a risk that the effects of between-group competition may be biased. Thus, it is necessary to investigate the pure effect of between-group competition on trust and trustworthiness in the future. Future research should also investigate the effects of within- and between-group competition on trust and trustworthiness among strangers.

## Supporting Information

Dataset S1 (ZIP)

## References

[1] Boyd R, Gintis H, Bowles S (2010) Coordinated punishment of defectors sustains cooperation and can proliferate when rare. Sci 328: 617–620. 10.1126/science.1183665 20431013

[2] De Dreu CK (2012) Oxytocin modulates cooperation within and competition between groups: An integrative review and research agenda. Horm Behav 61: 419–428. 22227278 10.1016/j.yhbeh.2011.12.009

[3] Gintis H, Bowles S, Boyd R, Fehr E (2003) Explaining altruistic behavior in humans. Evol Hum Behav 24: 153–172.

[4] Hruschka DJ, Henrich J (2006) Friendship, cliquishness, and the emergence of cooperation. J Theor Biol 239: 1–15. 16102781 10.1016/j.jtbi.2005.07.006

[5] West SA, Ei Mouden C, Gardner A (2011) Sixteen common misconceptions about the evolution of cooperation in humans. Evol Hum Behav 32: 231–262.

[6] West SA, Griffin AS, Gardner A (2007) Evolutionary explanations for cooperation. Curr Biol 17: 661–672. 10.1016/j.cub.2007.06.004 17714660

[7] Dasgupta P, Goyal S, Mäler KG, Putnam R, Serageldin I (2009) A matter of trust: Social capital and economic development. In Lin JY, Pleskovic B, editors. Lessons from East Asia and the global financial crisis. World Bank Publications. pp. 119–156.

[8] De Cremer D, Dewitte S (2002) Effect of trust and accountability in mixed-motive situations. J Soc Psychol 142: 541–543. 12153128 10.1080/00224540209603917

[9] Deutsch M (1962) Cooperation and trust: Some theoretical notes. In Jones M, editors. Nebraska Symposium on Motivation. Lincoln: University of Nebraska. pp: 275–320.

[10] Balliet D, Mulder LB, Van Lange PA (2011) Reward, punishment, and cooperation: A meta-analysis. Psychol Bull 137: 594–615. 21574679 10.1037/a0023489

[11] Fehr E, Gächter S (2002) Altruistic punishment in humans. Nat 415: 137–140. 10.1038/415137a 11805825

[12] Fernald RD (2011) Animal cooperation: Keeping a clean(ing) reputation. Curr Biol 21: 508–510. 21741588 10.1016/j.cub.2011.05.038

[13] Herrmann B, Thöni C, Gächter S (2008) Antisocial punishment across societies. Sci 319: 1362–1367. 10.1126/science.1153808 18323447

[14] King-Casas B, Tomlin D, Anen C, Camerer CF, Quartz SR, Montague PR (2005) Getting to know you: reputation and trust in a two-person economic exchange. Sci 308: 78–83. 10.1126/science.1108062 15802598

[15] Sylwester K, Roberts G (2010) Cooperators benefit through reputation-based partner choice in economic games. Biol Lett 6: 659–662. 20410026 10.1098/rsbl.2010.0209 PMC2936156

[16] Bolton G, Loebbecke C, Ockenfels A (2008) Does competition promote trust and trustworthiness in online trading? An experimental study. J Manag Inf Syst 25: 145–170.

[17] Huck S, Lünser GK, Tyran JR (2012) Competition fosters trust. Games Econ Behav 76: 195–209.

[18] Hogg MA, Abrams D (1988) Social identifications: A social psychology of intergroup relations and group processes. Florence, KY: Taylor & Frances/Routledge.

[19] Turner JC, Hogg MA, Oakes PJ, Reicher SD, Wetherell MS (1987) Rediscovering the social group: A self-categorization theory. London: Basil Blackwell.

[20] Burton-Chellew MN, Ross-Gillespie A, West SA (2010) Cooperation in humans: Competition between groups and proximate emotions. Evol Hum Behav 31: 104–108.

[21] De Dreu CK, Greer LL, Handgraaf MJ, Shalvi S, Van Kleef GA, et al (2010) The neuropeptide oxytocin regulates parochial altruism in intergroup conflict among humans. Sci 328: 1408–1411. 10.1126/science.1189047 20538951

[22] Erev I, Bornstein G, Galili R (1993) Constructive intergroup competition as a solution to the free rider problem: A field experiment. J Exp Soc Psychol 29: 463–478.

[23] Gunnthorsdottir A, Rapoport A (2006) Embedding social dilemmas in intergroup competition reduces free-riding. Organ Behav Hum Decis Process 101: 184–199.

[24] Rebers S, Koopmans R (2012) Altruistic punishment and between-group competition. Hum Nat 23: 173–190. 22623138 10.1007/s12110-012-9136-x PMC3387358

[25] Puurtinen M, Mappes T (2009) Between-group competition and human cooperation. Proc R Soc B Biol Sci 276: 355–360. 10.1098/rspb.2008.1060 PMC2581672 18826935

[26] Tan JH, Bolle F (2007) Team competition and the public goods game. Econ Lett 96: 133–139.

[27] Bowles S (2006) Group competition, reproductive leveling, and the evolution of human altruism. Sci 314: 1569–1572. 10.1126/science.1134829 17158320

[28] Wilson DS (1975) A theory of group selection. Proc Natl Acad Sci 72: 143–146. 1054490 10.1073/pnas.72.1.143 PMC432258

[29] Deutsch M (1949) An experimental study of the effects of cooperation and competition upon group process. Hum Relat 2: 199–231.

[30] Johnson DW, Johnson RT (1989) Cooperation and competition: Theory and research. Edina, MN: Interaction.

[31] Johnson DW, Johnson RT (2005) New development in social interdependence theory. Genet Soc Gen Psychol Monogr 131: 285–358. 17191373 10.3200/MONO.131.4.285-358

[32] Rosenbaum ME, Moore DL, Cotton JL, Cook MS, Hieser RA, et al (1980) Group productivity and process: Pure and mixed reward structures and task interdependence. J Pers Soc Psychol 39: 626–642.

[33] Barker JL, Barclay P, Reeve HK (2012) Within-group competition reduces cooperation and payoffs in human groups. Behav Ecol 23: 735–741.

[34] De Dreu CK, Weingart LR (2003) Task versus relationship conflict, team performance, and team member satisfaction: A meta-analysis. J Appl Psychol 88: 741–749. 12940412 10.1037/0021-9010.88.4.741

[35] Ruscher JB, Fiske ST, Mikl H, Van Manen S (1991) Individuating processes in competition: Interpersonal versus intergroup. Pers Soc Psychol Bull 17: 595–605.

[36] Nowak MA, Sigmund K (2005) Evolution of indirect reciprocity. Nat 437: 1291–1298. 10.1038/nature04131 16251955

[37] Riolo RL, Cohen MD, Axelrod R (2001) Evolution of cooperation without reciprocity. Nat 414: 441–443. 10.1038/35106555 11719803

[38] Sigmund K (2012) Moral assessment in indirect reciprocity. J Theor Biol 299: 25–30. 21473870 10.1016/j.jtbi.2011.03.024 PMC3334524

[39] Wedekind C, Milinski M (2000) Cooperation through image scoring in humans. Sci 288: 850–852. 10.1126/science.288.5467.850 10797005

[40] Charness G, Du N, Yang CL (2011) Trust and trustworthiness reputations in an investment game. Games Econ Behav 72: 361–375.

[41] Berg J, Dickhaut J, McCabe K (1995) Trust, reciprocity, and social history. Games Econ Behav 10: 122–142.

[42] Burks SV, Carpenter JP, Verhoogen E (2003) Playing both roles in the trust game. J Econ Behav Organ 51: 195–216.

[43] Chaudhuri A, Gangadharan L (2007) An experimental analysis of trust and trustworthiness. South Econ J 73: 959–985.

[44] Croson R, Buchan N (1999) Gender and culture: International experimental evidence from trust games. Am Econ Rev 89: 386–391.

[45] West SA, Gardner A, Shuker DM, Reynolds T, Burton-Chellow M, et al (2006) Cooperation and the scale of competition in humans. Curr Biol 16: 1103–1106. 16753564 10.1016/j.cub.2006.03.069

[46] Cikara M, Botvinick MM, Fiske ST (2011) Us versus them: Social identity shapes neural responses to intergroup competition and harm. Psychol Sci 22: 306–313. 21270447 10.1177/0956797610397667 PMC3833634

[47] Tajfel H (1981) Social identity and intergroup relations. London: Cambridge University Press.

[48] Tajfel H, Billig MG, Bundy RP, Flament C (1971) Social categorization and intergroup behaviour. Eur J Soc Psychol 1: 149–178.

[49] Johnson DW, Maruyama G, Johnson R, Nelson D, Skon L (1981) Effects of cooperative, competitive, and individualistic goal structures on achievement: A meta-analysis. Psychol Bull 89: 47–62.

[50] Rabbie JM, Horwitz M (1969) Arousal of ingroup-outgroup bias by a chance win or loss. J Pers Soc Psychol 13: 269–277. 5352845 10.1037/h0028284

[51] Sharan S (1990) Cooperative learning: Theory and research. New York: Praeger.

[52] Niu J, Xin Z, Martins N (2010) Trust discrimination tendency in average citizens at in-nation and out-nation level in Canada, China and the United States. Int J Psychol Stud 2: 12–24.

[53] Mulder LB, Van Dijk E, De Cremer D, Wilke HAM (2006) Undermining trust and cooperation: The paradox of sanctioning systems in social dilemmas. J Exp Soc Psychol 42: 147–162.

