# Supplementary material for: The Effects of Within- and Between-Group Competition on Trust and Trustworthiness among Acquaintances
Source: PLoS One. 2014 Jul 18;9(7):e103074. doi: 10.1371/journal.pone.0103074 (PMC4103862; doi:10.1371/journal.pone.0103074)
Supplement: Dataset S1 — (ZIP) [file pone.0103074.s001.zip › instructions for datasets.doc]

The variables in “trustor.sav” were interpreted following:

**Gender**: 1=female; 2=male

**Age**: participants’ age

**Investone**: trustor’s investment in round one

**Investtwo**: trustor’s investment in round two

……

**Investthirteen**: trustor’s investment in round thirteen

**Returnone**: the money that trustor received from trustee in round one

**Returntwo**: the money that trustor received from trustee in round two

……

**Returnthirteen**: the money that trustor received from trustee in round thirteen

**Profitone**: trustor’s payoff in round one which equals 10-investone+returnone

**Profittwo**: trustor’s payoff in round two which equals 10-investtwo+returntwo

……

**Profitthirteen**: trustor’s payoff in round thirteen which equals 10-investthirteen+returnthirteen

**Meaninvestone**: trustor’s average investment in phase one which equals (investone+investtwo+investthree)/3

**Meaninvesttwo**: trustor’s average investment in phase two which equals (investfour+investfive+investsix+investseven+investeight)/5

**Meaninvestthree**: trustor’s average investment in phase three which equals (investnine+investten+investeleven+investtwelve+investthirteen)/5

**Meanprofitone**: trustor’s average payoff in phase one which equals (profitone+profittwo+profitthree)/3

**Meanprofittwo**: trustor’s average payoff in phase two which equals (profitfour+profitfive+profitsix+profitseven+profiteight)/3

**Meanprofitthree**: trustor’s average payoff in phase three which equals (profitnine+profitten+profiteleven+profittwelve+profitthirteen)/3

The variables in “trustee.sav” were interpreted following:

**Gender**: 1=female; 2=male

**Age**: participants’ age

**acceptedone**: the money trustee accepted from trustor in round one which equals three times of the investment of their corresponding trustor

**acceptedtwo**: the money trustee accepted from trustor in round two which equals three times of the investment of their corresponding trustor

……

**acceptedthirteen**: the money trustee accepted from trustor in round thirteen which equals three times of the investment of their corresponding trustor

**Returnone**: the money that trustee returned to their corresponding trustor in round one

**Returntwo**: the money that trustee returned to their corresponding trustor in round two

……

**Returnthirteen**: the money that trustee returned to their corresponding trustor in round thirteen

**Profitone**: trustee’s payoff in round one which equals 10+acceptedone-returnone

**Profittwo**: trustee’s payoff in round two which equals 10+acceptedtwo-returntwo

……

**Profitthirteen**: trustee’s payoff in round thirteen which equals 10+acceptedthirteen-returnthirteen

**Ratioone**: trustee’s return ratio in round one which equals returnone/acceptedone

**Ratiotwo**: trustee’s return ratio in round two which equals returntwo/acceptedtwo

……

**Ratiothirteen**: trustee’s return ratio in round thirteen which equals returnthirteen/acceptedthirteen

**Meanratioone**: trustee’s average return ratio in phase one which equals (ratioone+ratiotwo+ratiothree)/3

**Meanratiotwo**: trustee’s average return ratio in phase two which equals (ratiofour+ratiofive+ratiosix+ratioseven+ratioeight)/5

**Meanratiothree**: trustee’s average return ratio in phase three which equals (rationine+ ratioten+ ratioeleven+ ratiotwelve+ ratiothirteen)/5

**Meanprofitone**: trustee’s average payoff in phase one which equals (profitone+profittwo+profitthree)/3

**Meanprofittwo**: trustee’s average payoff in phase two which equals (profitfour+profitfive+profitsix+profitseven+profiteight)/3

**Meanprofitthree**: trustee’s average payoff in phase three which equals (profitnine+profitten+profiteleven+profittwelve+profitthirteen)/3
